# Serological and Clinical Evaluation of Incompatible Crossmatches Encountered in Pre-transfusion Testing

**DOI:** 10.7759/cureus.101149

**Published:** 2026-01-09

**Authors:** Anisha Badoni, Anuradha Kusum, Manish Raturi

**Affiliations:** 1 Department of Pathology, Himalayan Institute of Medical Sciences, Dehradun, IND; 2 Department of Immunohematology and Blood Transfusion, Himalayan Institute of Medical Sciences, Dehradun, IND

**Keywords:** alloantibody, autoantibody, daratumumab interference, incompatible crossmatch, pre-transfusion testing

## Abstract

Background

Pre-transfusion testing is essential for preventing incompatible red cell transfusions and associated adverse reactions. Despite modern serological advancements, unexpected alloantibodies, autoantibodies, and monoclonal antibody interference continue to contribute to incompatible crossmatches. This study evaluates the serological patterns, clinical associations, and diagnostic considerations of incompatible crossmatches encountered in routine transfusion practice.

Methods

This cross-sectional study was conducted at a tertiary-care transfusion service of Swami Rama Himalayan University, Dehradun, India, over a duration of 18 months, from April 2023 to September 2024. Of 34,631 packed red blood cell (PRBC) crossmatches performed, 46 (0.13%) showing major incompatibility were included. Detailed clinical history, hematological parameters, and immunohematological testing, including Direct Coombs Test (DCT), Indirect Coombs Test (ICT), extended antibody panels, and daratumumab interference assessment, were performed. Associations between antibody category and clinical variables were analyzed using Chi-square/Fisher’s exact tests, with p < 0.05 considered significant.

Results

Among 46 incompatible crossmatches, alloantibodies were the most frequent antibody type (24; 52.1%), followed by autoantibodies (16; 34.8%) and daratumumab interference (6; 13.0%). DCT status showed a significant association with antibody type (p = 0.001), with autoantibodies observed exclusively in DCT-positive cases (16/16; 100%), while daratumumab interference occurred only in DCT-negative cases (6/6; 100%). Significant associations were observed for age group (p = 0.012), clinical diagnosis (p = 0.001), previous transfusion history (p = 0.001), number of PRBC units transfused (p = 0.001), hepatomegaly (p = 0.009), and splenomegaly (p = 0.001). Younger patients predominantly exhibited alloantibodies, whereas patients older than 50 years showed contributions from all antibody categories. Autoantibodies predominated in benign hematological disorders (12; 75.0%), while daratumumab interference was confined to hematological malignancies (6; 100%). Gender (p = 0.463) and Rh typing (p = 0.076) were not significantly associated with antibody distribution. Logistic regression analysis did not identify reliable independent predictors due to wide confidence intervals and model instability.

Conclusion

Most incompatible crossmatches were caused by alloantibodies, though clinically significant contributions from autoantibodies and daratumumab interference were also observed. Strong associations with demographic and diagnostic variables highlight the importance of individualized immunohematological assessment. Routine use of structured diagnostic algorithms can enhance resolution of incompatible crossmatches and strengthen transfusion safety.

## Introduction

The field of transfusion medicine has evolved from basic serological techniques to sophisticated molecular approaches, reflecting significant scientific progress and continuous innovation in healthcare, particularly in India [1]. Historically, bloodletting or venesection was a dominant therapeutic practice from the era of Hippocrates (430 BC) until the nineteenth century. However, blood transfusion became widespread only in the past century due to earlier limitations in the understanding of blood physiology and circulation. Prior to the scientific developments of the mid-17th century, medical practice was shaped largely by Roman and Greek theories, which guided healthcare for nearly two millennia [2].

Pretransfusion testing remains fundamental to safe transfusion practice [3]. Its primary goal is to ensure compatibility between donor and recipient by preventing adverse reactions and optimizing the survival of transfused red blood cells (RBCs). This involves ABO and Rh blood grouping, screening for unexpected alloantibodies, antibody identification when screens are positive, careful selection of appropriate blood components, and compatibility testing through crossmatching [4]. These steps collectively minimize risks and form the backbone of modern transfusion safety.

Despite its indispensable role, blood transfusion still carries risks, particularly adverse transfusion reactions (ATRs). Acute hemolytic transfusion reactions (AHTRs), often caused by ABO-incompatible (ABOi) transfusions, remain among the most severe [5]. The true burden of ABOi transfusions may be underestimated due to inconsistent reporting practices. Current clinical estimates place their frequency between 1 in 10,000 and 1 in 4,667 units, though actual numbers may be higher. Accurate reporting and detailed analysis of ATRs are essential to understanding causative factors and strengthening preventive measures [5].

Pretransfusion testing significantly reduces incompatibility-related events, yet it cannot entirely eliminate risk. Rare antibodies or low-frequency antigens may remain undetected, and transfusion of such units can lead to suboptimal RBC survival or unexpected reactions [6]. Even when ABO and Rh compatibility is ensured, other clinically significant antigens can cause alloimmunization. For this reason, crossmatching remains essential, allowing direct assessment of donor RBC compatibility with the recipient’s serum. In certain cases, even after exhaustive testing, fully compatible units may not be available, necessitating reliance on the "least incompatible" blood. Such decisions require coordinated judgment between clinicians and transfusion medicine specialists [7].

The need for this study arises from the continuing challenges in ensuring uncompromised transfusion safety. Incompatible crossmatches result primarily from unexpected antibodies against less common antigens, which may not be detected by routine testing but can precipitate hemolytic reactions [8]. A systematic evaluation of these incompatibilities will enhance understanding of antibody-antigen interactions and contribute to improved diagnostic accuracy [9]. Furthermore, the substantial variability in antigen expression - both among individuals and under varying physiological conditions - can complicate crossmatching. Understanding these variations may guide the development of refined testing algorithms.

Current serological tools, though effective in most scenarios, face limitations in detecting multiple or low-frequency antibodies. Improved methods are especially important for chronically transfused patients, who are prone to developing alloantibodies that complicate future transfusions. By identifying the antibodies responsible for incompatible crossmatches, this research may support better patient management strategies and help reduce alloimmunization.

A deeper understanding of the mechanisms underlying incompatible crossmatches, coupled with advancements in serological testing, is vital for enhancing transfusion safety. The objectives of the study were to identify the serological causes of incompatible crossmatches, to evaluate the association of different antibody categories with clinical and transfusion-related variables, and to assess the diagnostic patterns used in resolving incompatible crossmatches.

## Materials and methods

Study setting and duration

This study was conducted in the Department of Pathology in collaboration with the Department of Immunohematology and Blood Transfusion at Swami Rama Himalayan University, Swami Ram Nagar, Dehradun. The study duration was 18 months, from April 2023 to September 2024. Ethical clearance was obtained from the institutional ethics committee, and informed written consent was taken from all participating patients. Samples were collected from blood units submitted to the blood center for crossmatching. Cases showing mixed-field agglutination reactions during conventional serologic crossmatching were selected for further analysis. Additional assessment of blood typing was performed to evaluate agglutination patterns reflecting membrane-bound agglutinogens. The study followed a cross-sectional design and employed consecutive purposive convenience sampling.

Sample size calculation

The minimum sample size was derived using the formula:

$n = \frac{Z^{2}_{(1-\alpha/2)} \times P \times (1 - P)}{d^{2}}$,

where Z = 1.96 at a 95% confidence interval, P = prevalence of incompatible crossmatches (0.69%) as per Bhattacharya et al. [10], and d = 2.5% allowable error. The calculated sample size was 42, and assuming a 10% dropout rate, a minimum of 46 patients was required.

Selection of participants

The study included all inpatients whose blood samples demonstrated major incompatible crossmatches during routine pre-transfusion testing, irrespective of the presence or absence of mixed-field agglutination. All included cases showed positive Indirect Coombs Test (ICT) results, which formed part of the serological profile of incompatible crossmatches evaluated in this study.

Study tools and materials

Data collection utilized a case recording form and requisition forms. Laboratory materials included gel cards for crossmatching, transfusion consent forms, blood grouping setups using the tube technique, and 2-3 mL properly labelled ethylenediaminetetraacetic acid (EDTA) and plain tube samples.

Study protocol

Baseline patient characteristics, including name, age, gender, weight, Unique Health Identification (UHID) number, contact details, blood group, and hemoglobin, were documented in a case recording form for patients requiring transfusion and meeting inclusion criteria. Written consent for transfusion was obtained. A detailed clinical history was taken, covering medication use, prior transfusions, pregnancy or abortion history in females, and any previous transfusion reactions. Each transfusion request was accompanied by 2-3 mL of correctly labelled EDTA and plain tube samples. Blood grouping was carried out using the conventional tube technique with monoclonal antisera, and reverse grouping was employed with in-house prepared pooled A, B, and O cells validated by saline control. Crossmatching and antibody screening were performed using column agglutination technology (CAT) gel cards, following the manufacturer’s instructions. The platform utilized standardized incubation conditions, centrifugation parameters, and reagent volumes to ensure reproducibility of serological results. In brief, 10 μL of 4% donor RBC suspension, 40 μL patient serum, and 50 μL of bliss reagent were added, incubated at 37°C for 15 minutes, and centrifuged for 5 minutes. In cases of incompatible crossmatches, further immunohematology (IH) workup was performed using 3-cell and 11-cell extended antibody identification panels to determine the specific erythrocyte antibody present in the serum. Persistently incompatible crossmatches underwent a stepwise immunohematological evaluation, including DCT, ICT, extended antibody identification panels, and DTT treatment when indicated, following a standardized institutional algorithm.

Definitions

Anemia severity was classified based on hemoglobin levels as mild (10-10.9 g/dL), moderate (7-9.9 g/dL), and severe (<7 g/dL), in accordance with standard clinical practice. Hepatomegaly and splenomegaly were assessed based on clinical examination findings documented in patient records and corroborated by ultrasonography or other imaging modalities when available.

Data management and statistical analysis

Data entry was performed using Microsoft Excel (Microsoft Corporation, Redmond, WA), and statistical analysis was conducted using SPSS version 26 (IBM Corp., Armonk, NY). Categorical variables were summarized as frequencies and percentages, while quantitative variables were expressed as mean ± standard deviation. Group comparisons were performed using a t-test for continuous variables and a chi-square or Fisher’s exact test for categorical variables. Multivariable analysis was carried out using logistic regression models to evaluate predictors of antibody type and incompatible crossmatches. A p-value < 0.05 was considered statistically significant.

## Results

Out of 34,631 packed RBC (PRBC) crossmatches performed during the study period, 46 cases (0.13%) were found to be incompatible and included in the analysis. The mean age of the study population was 40.8 ± 11.2 years, with a higher proportion of cases occurring at the extremes of age, particularly in patients older than 50 years (41.3%). Females accounted for 67.4% of incompatible crossmatches. Most patients had moderate (28.3%) to severe anemia (32.6%), with transfusion being more frequently required in these categories compared to mild anemia (2.2%). ICT was positive in all included cases (100%), reflecting the presence of unexpected antibodies in patients with incompatible crossmatches. The Direct Coombs Test (DCT) showed variable reactivity, being positive in 41.3% of patients. Detailed demographic characteristics, anemia severity, transfusion requirements, and Coombs test findings are presented in Table 1.

**Table 1:** Distribution of demographic, clinical, and serological parameters among patients with incompatible crossmatches. DCT: Direct Coombs Test; ICT: Indirect Coombs Test; PRBC: packed red blood cells

Parameter	Domain	N (%)
Age distribution	<10 years	9 (19.6%)
	10-20 years	3 (6.5%)
	21-30 years	6 (13.0%)
	31-40 years	5 (10.9%)
	41-50 years	4 (8.7%)
	>50 years	19 (41.3%)
Gender distribution	Male	15 (32.6%)
	Female	31 (67.4%)
Transfusion requirement by anemia severity	Mild anemia - required	1 (2.2%)
	Mild anemia - not required	3 (6.5%)
	Moderate anemia - required	13 (28.3%)
	Moderate anemia - not required	9 (19.5%)
	Severe anemia - required	15 (32.6%)
	Severe anemia - not required	5 (10.9%)
DCT results	Positive	19 (41.3%)
	Negative	11 (23.9%)
	Not done	16 (34.8%)
ICT results	Positive	46 (100%)

Among patients with incompatible crossmatches, alloantibodies were the most frequently identified antibody type, accounting for 52.1% of cases, followed by autoantibodies at 34.8%, while daratumumab-related interference constituted 13.0%. A statistically significant association was observed between antibody type and DCT status (p = 0.001). Autoantibodies were present exclusively among DCT-positive cases at 100%, whereas alloantibodies were more commonly identified in DCT-negative cases at 41.7% and in cases where DCT was not performed at 37.5% (Table 2).

**Table 2:** Distribution of alloantibody, autoantibody, and daratumumab influence across clinical and laboratory variables. p-values were calculated using the chi-square test. A p-value < 0.05 was considered statistically significant.

Variable	Category	Daratumumab (n = 6)	Alloantibody (n = 24)	Autoantibody (n = 16)	Chi-square Statistics	p-Value
DCT result	Not done	0	9 (37.5%)	0	37.81	0.001*
	Positive	0	5 (20.8%)	16 (100.0%)		
	Negative	6 (100.0%)	10 (41.7%)	0		
Gender	Male	2 (33.3%)	6 (25.0%)	7 (43.8%)	1.53	0.463
	Female	4 (66.7%)	18 (75.0%)	9 (56.2%)		
Age group	<10 years	0	5 (20.8%)	4 (25.0%)	22.51	0.012*
	10-20	0	1 (4.2%)	2 (12.5%)		
	21-30	0	6 (25.0%)	0		
	31-40	0	5 (20.8%)	0		
	41-50	0	1 (4.2%)	3 (18.8%)		
	>50	6 (100.0%)	6 (25.0%)	7 (43.8%)		
Clinical diagnosis	Acute febrile illness	0	10 (41.7%)	2 (12.5%)	37.64	0.001*
	Antenatal anemia	0	3 (12.5%)	0		
	Benign hematological disorder	0	4 (16.7%)	12 (75.0%)		
	Hematological malignancies	6 (100.0%)	4 (16.7%)	1 (6.2%)		
	Trauma and hemorrhage	0	3 (12.5%)	1 (6.2%)		
Previous transfusion	No	4 (66.7%)	5 (20.8%)	14 (87.5%)	17.83	0.001*
	Yes	2 (33.3%)	19 (79.2%)	2 (12.5%)		
Number of PRBC units	None	4 (66.7%)	5 (20.8%)	14 (87.5%)	18.50	0.001*
	1-3 units	2 (33.3%)	15 (62.5%)	2 (12.5%)		
	4-6 units	0	4 (16.7%)	0		
Rh typing	Rh negative	0	5 (20.8%)	0	5.14	0.076
	Rh positive	6 (100.0%)	19 (79.2%)	16 (100.0%)		
Hepatomegaly	Absent	6 (100.0%)	22 (91.7%)	9 (56.3%)	9.33	0.009*
	Present	0	2 (8.3%)	7 (43.7%)		
Splenomegaly	Absent	6 (100.0%)	23 (95.8%)	8 (50.0%)	14.49	0.001*
	Present	0	1 (4.2%)	8 (50.0%)		

Gender distribution did not demonstrate a statistically significant association with antibody type (p = 0.463), despite females comprising 67.4% of the study population and exhibiting a higher proportion of alloantibodies at 75.0% compared to males, in whom autoantibodies were relatively more common at 43.8%. Antibody distribution differed significantly across age groups (p = 0.012). Patients younger than 40 years predominantly exhibited alloantibodies, accounting for approximately two-thirds of cases in these age groups, while patients older than 50 years showed contributions from all antibody categories, including daratumumab-related interference at 100% within that age group (Table 2).

Clinical diagnosis showed a strong and statistically significant association with antibody type (p = 0.001). Alloantibodies were most frequently observed in acute febrile illness at 41.7%, antenatal anemia at 12.5%, and trauma or hemorrhage at 12.5%. Autoantibodies predominated in benign hematological disorders at 75.0%, whereas daratumumab-related interference was confined to patients with hematological malignancies at 100%. A significant association was identified between prior transfusion history and antibody type (p = 0.001). Among previously transfused patients, alloantibodies were present in 79.2% of cases, while autoantibodies predominated among non-transfused patients at 87.5%. The number of PRBC units transfused also demonstrated a significant association (p = 0.001), with alloantibodies observed in 62.5% of patients receiving one to three units and 16.7% of those receiving four to six units (Table 2).

Rh blood group status did not show a statistically significant relationship with antibody distribution (p = 0.076), although all autoantibodies and daratumumab-related cases occurred in Rh-positive patients. Among clinical examination findings, hepatomegaly and splenomegaly demonstrated significant associations with antibody type. Autoantibodies were more frequently observed in patients with hepatomegaly at 43.7% and splenomegaly at 50.0%, whereas alloantibodies predominated in patients without hepatomegaly at 91.7% and without splenomegaly at 95.8% (Table 2).

Multivariable regression analysis assessing predictors of antibody type did not identify any statistically significant associations. All evaluated variables, including gender, age group, clinical diagnosis, and prior transfusion history, demonstrated non-significant relationships, characterized by very high p-values and extremely wide confidence intervals (Table 3). Although some predictors yielded large odds ratio estimates, these were accompanied by wide and imprecise confidence intervals, indicating substantial model instability rather than true clinical associations. Overall, the regression model failed to reliably explain variations in antibody type, and the findings should therefore be interpreted with caution (Table 3).

**Table 3:** Logistic regression analysis of predictors associated with antibody type (daratumumab influence, alloantibody, and autoantibody).

Predictor	Estimate	SE	Z	p-Value	Odds Ratio	95% CI (Lower to Upper)
Intercept	-64.295	39421	-0.00	0.999	1.19	-77328 to 77199
Gender (female vs. male)	21.053	15996	0.00	0.999	1.39	-31330 to 31372
Age (10-20 vs. <10)	0.712	68601	1.04	1.000	2.04	-134456 to 134457
Age (21-30 vs. <10)	-22.045	25610	-8.61	0.999	2.67	-50217 to 50172
Age (31-40 vs. <10)	-21.533	27893	-7.72	0.999	4.45	-54691 to 54648
Age (41-50 vs. <10)	21.765	90106	2.42	1.000	2.83	-176583 to 176627
Age (>50 vs. <10)	62.763	33091	0.00	0.998	1.81	-64794 to 64920
Diagnosis (antenatal anemia vs. AFI)	66.095	28043	0.00	0.001	6.02	-94652 to 94785
Diagnosis (benign hematologic disorder vs. AFI)	43.241	38357	0.00	0.001	2.24	-54920 to 55007
Diagnosis (hematologic malignancy vs. AFI)	65.279	124688	6.96	0.0017	4.74	-75113 to 75243
Diagnosis (trauma/hemorrhage vs. AFI)	86.752	88703	4.59	0.006	4.88	-244298 to 244471
Previous transfusion (yes vs. no)	-41.709	28968	0.00	0.999	7.69	-56818 to 56735

The logistic regression model using alloantibody as the reference outcome did not identify any meaningful or statistically significant predictors. Nearly all p-values approached 1, and estimated odds ratios for clinical variables, including splenomegaly, hepatomegaly, anemia severity, transfusion history, Rh typing, and number of transfused units, showed extreme variability with excessively wide confidence intervals, indicating poor model stability (Table 4). Although some variables demonstrated large odds ratio estimates, such as severe anemia compared to mild anemia and receipt of two units compared to no transfusion, these findings were accompanied by large standard errors and wide confidence intervals and therefore lacked statistical reliability. Overall, the analysis indicates that none of the evaluated clinical, laboratory, or transfusion-related variables independently predicted the development of specific antibody types (Table 4).

**Table 4:** Logistic regression model assessing clinical and laboratory predictors of outcome using alloantibody as reference.

Predictor	Estimate	SE	Z	p-Value	Odds Ratio	95% CI (Lower to Upper)
Intercept	-127.093	23717.55	-0.00536	0.996	6.37	-46612.65 to 46358.46
Rh typing (+ vs. -)	90.931	17200.73	0.00529	0.996	3.10	-33621.88 to 33803.74
Previous transfusion (yes vs. no)	-18.842	5555.72	-0.00339	0.997	6.56	-10907.86 to 10870.17
1 unit transfused vs. none	19.803	5555.72	0.00356	0.997	3.99	-10869.21 to 10908.82
2 units transfused vs. none	20.565	5555.72	0.00370	0.997	8.54	-10868.45 to 10909.58
3 units transfused vs. none	21.657	5555.72	0.00390	0.997	2.55	-10867.36 to 10910.67
4 units transfused vs. none	40.166	29755.70	0.00135	0.999	2.78	-58279.93 to 58360.26
6 units transfused vs. none	14.459	234872.66	6.16	1.000	1.90	-460327.49 to 460356.41
7 units transfused vs. none	40.166	29755.70	0.00135	0.999	2.78	-58279.93 to 58360.26
Hepatomegaly (present vs. absent)	54.193	10809.36	0.00501	0.996	3.43	-21131.76 to 21240.15
Splenomegaly (present vs. absent)	-0.373	2.07	-0.17968	0.857	0.6888	-4.44 to 3.694
DCT positive vs. not done	0.666	1.57	0.42442	0.671	1.9473	-2.41 to 3.744
DCT negative vs. not done	-2.343	1.57	-1.49462	0.135	0.0961	-5.41 to 0.729
Moderate vs. mild anemia	35.737	8265.72	0.00432	0.997	3.31	-16164.78 to 16236.25
Severe vs. mild anemia	34.31	8265.72	0.00415	0.997	7.95	-16166.21 to 16234.83

**Figure 1:**
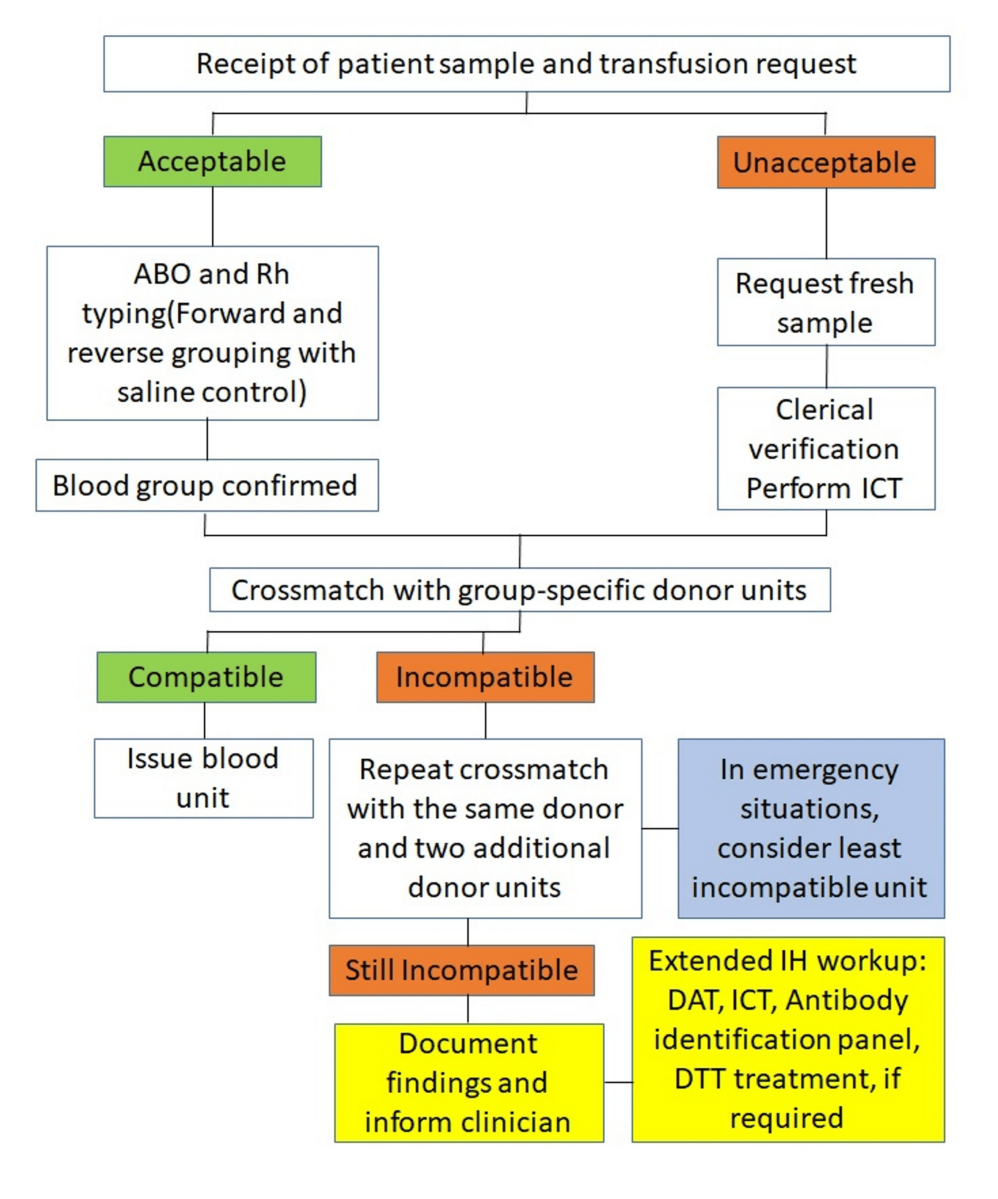
Standardized workflow for resolving incompatible crossmatches in the transfusion service.

A standardized algorithm was followed for resolving incompatible crossmatches in the blood center. Each patient sample underwent initial clerical and serological verification, including ABO and Rh typing with forward and reverse grouping. Samples deemed unacceptable were re-collected, while discrepancies triggered repeat testing and clinical history review. Crossmatching with group-specific donor units served as the primary compatibility check. Compatible units were issued immediately, whereas incompatible reactions prompted repeat crossmatching with additional donor units. Persistently incompatible cases underwent extended IH work-up, including direct antiglobulin test (DAT), ICT, antibody identification panels, and dithiothreitol (DTT) treatment when indicated. In life-threatening situations, the least incompatible unit was considered with the clinician's consent. All steps and outcomes were documented to ensure traceability and transfusion safety (Figure 1).

## Discussion

This study evaluated the prevalence and underlying causes of incompatible crossmatches in a tertiary care transfusion service. In our study, incompatible crossmatches represented a small proportion of routine pretransfusion testing, providing objective insight into the burden of serological discrepancies encountered in day-to-day transfusion practice. The study population included a broad age range, with a higher representation of older individuals, and females constituted a greater proportion of cases. This observation is consistent with previous reports suggesting that female patients, due to pregnancy-related antigen exposure, more frequently develop RBC antibodies [11].

The overall antibody distribution demonstrated that alloantibodies were the most common cause of incompatibility, followed by autoantibodies, with daratumumab-related interference accounting for a smaller but clinically important proportion. This pattern is comparable to earlier studies reporting alloantibody predominance in incompatible crossmatches, with reported frequencies ranging from approximately 50 to 70% [10,11]. Daratumumab interference emerged as a notable contributor, particularly among patients with hematological malignancies, consistent with recent reports describing anti-CD38 therapeutic interference in serologic testing, where rates of panreactivity have been reported in up to 100% of treated patients [12].

DCT results demonstrated distinct antibody profiles. Autoantibodies were exclusively associated with DCT positivity, whereas alloantibodies were more frequently observed among DCT-negative cases and in those where the test was not performed. Daratumumab-related interference was confined to DCT-negative cases. The statistically significant association between DCT status and antibody type highlights the diagnostic utility of this test in distinguishing alloimmune from autoimmune mechanisms of incompatibility. Similar observations were made by Vidushi et al. [11] and Oberman et al. [13], who emphasized the importance of structured immunohematological algorithms in resolving complex serologic patterns.

Gender did not demonstrate an independent statistically significant association with antibody distribution in the present analysis. Although previous studies, such as Bhattacharya et al. [10], reported higher alloimmunization rates among females, attributing this to pregnancy-related antigen exposure, other studies have similarly failed to demonstrate a consistent gender effect after accounting for exposure-related variables. This suggests that observed gender differences may be influenced more by clinical context than by intrinsic biological susceptibility.

Age group showed a statistically significant association with antibody distribution. Prior studies have reported varying age-related trends, with Bhattacharya et al. [10] describing higher incompatibility rates among younger adults, while Vidushi et al. [11] observed increased complexity in older patients due to cumulative transfusion exposure. The findings of the present study align with this variability and underscore the influence of population-specific and disease-related factors on antibody formation.

Clinical diagnosis demonstrated a strong and statistically significant association with antibody type. Autoantibodies predominated in benign hematological disorders, a finding consistent with Shoganraj et al. [14], who reported autoantibody frequencies exceeding 70% in immune-mediated hematological conditions. The restriction of daratumumab-related interference to patients with hematological malignancies is in agreement with published evidence linking monoclonal antibody therapy to predictable serologic interference patterns [12].

Transfusion-related variables showed significant associations with antibody distribution. Previous studies have consistently demonstrated an increased risk of alloimmunization with prior transfusion exposure and higher cumulative RBC transfusion burden, with reported alloantibody rates increasing proportionally with transfusion frequency [11,15]. The present findings support these established mechanisms and reinforce the importance of judicious transfusion practices and extended antigen matching in selected patient populations.

Rh typing did not demonstrate a statistically significant association with antibody patterns in the present study. Although Bhattacharya et al. [10] reported a higher prevalence of alloantibodies among Rh-negative individuals, other investigators have noted that Rh status alone does not independently predict antibody formation in the absence of repeated antigen exposure [11].

Organomegaly demonstrated clinically relevant associations with antibody patterns. Prior studies have described an association between splenomegaly and autoimmune hemolytic anemia, with autoantibody-mediated hemolysis reported in up to 60% of patients with splenic enlargement [14]. The findings of the present study are consistent with these observations and support the role of the spleen in immune-mediated red cell destruction. Hepatomegaly has similarly been described as a marker of underlying hematological or systemic disease rather than a direct cause of antibody formation [10].

Overall, this study provides clinically relevant insights into the serologic characteristics of incompatible crossmatches encountered in routine transfusion practice. The significant associations observed with DCT status, age group, clinical diagnosis, transfusion-related variables, and organomegaly emphasize the importance of individualized immunohematological assessment. Conversely, the lack of significant association with gender and Rh typing highlights the multifactorial nature of antibody formation. These findings support the continued refinement of compatibility testing algorithms to enhance transfusion safety.

A key strength of this study is its conduct in a real-world tertiary care transfusion service, which reflects routine clinical workflows and operational challenges encountered during pre-transfusion testing. The use of a standardized, stepwise immunohematologic work-up, including direct and indirect Coombs testing, extended antibody identification panels, and assessment for daratumumab-related interference, strengthens the clinical relevance of the findings and supports their applicability to day-to-day transfusion decision-making.

The limitations of the study have been explicitly addressed and expanded in this revised Discussion. The relatively small sample size of incompatible crossmatches limited the statistical power of subgroup analyses and resulted in underpowered multivariable regression models with wide confidence intervals and unstable estimates. Consequently, the regression analysis could not reliably identify independent predictors and was interpreted descriptively rather than inferentially. In addition, antibody classification was based on available serologic techniques without routine molecular or confirmatory testing, introducing a potential risk of serologic misclassification, particularly in cases involving complex or low-frequency antibodies. Furthermore, as this was a single-center study with case selection restricted to patients undergoing crossmatch testing at one institution, the generalizability of the findings to other transfusion settings or populations may be limited.

Despite these limitations, the study offers valuable real-world insight into the serological patterns and clinical contexts of incompatible crossmatches and underscores the importance of structured diagnostic workflows in strengthening transfusion safety and guiding evidence-based transfusion practices.

## Conclusions

This study highlights the serological complexity underlying incompatible crossmatches and emphasizes the importance of detailed immunohematological evaluation in routine transfusion practice. Alloantibodies emerged as the most common cause of incompatibility, while autoantibodies and daratumumab-related interference contributed substantially in specific clinical settings. Significant associations with age, underlying clinical diagnosis, transfusion-related factors, and splenomegaly underscore the influence of patient and disease-related characteristics on antibody formation. Although multivariable logistic regression did not identify reliable independent predictors, the descriptive and univariable findings reinforce the need for a systematic, algorithm-based approach to resolving serological incompatibilities. Strengthening pretransfusion testing workflows and maintaining awareness of disease and therapy-related interference are essential to enhance transfusion safety and support evidence-based patient management.
